# TUNA‐EBSD‐CL correlative multi‐microscopy study, on the example of Cu(In,Ga)S_2_ solar cell absorber

**DOI:** 10.1111/jmi.13393

**Published:** 2025-02-02

**Authors:** Yucheng Hu, Gunnar Kusch, Damilola Adeleye, Susanne Siebentritt, Rachel Oliver

**Affiliations:** ^1^ Department of Materials Science and Metallurgy University of Cambridge Cambridge UK; ^2^ Laboratory for Photovoltaics Department of Physics and Materials Science Research Unit University of Luxembourg Belvaux Luxembourg

**Keywords:** cathodoluminescence, conductive‐AFM, electron backscatter diffraction, grain boundary, solar cell

## Abstract

Multi‐microscopy offers significant benefits to the understanding of complex materials behaviour by providing complementary information from different properties. However, some characterisations may strongly influence other measurements in the same workflow. To acquire reliable and valid datasets, optimising multi‐microscopy procedure is necessary. In present work, we studied the influence of the measurement order on the quality of multi‐microscopy datasets. Multi‐microscopy incorporating tunnelling current AFM (TUNA), electron backscatter diffraction (EBSD), and cathodoluminescence (CL) on a polycrystalline solar cell absorber, Cu(In,Ga)S_2_ (CIGS), is used as an example. The investigation revealed potential characterisation‐induced contaminations, such as surface oxidation and hydrocarbon layer coating, of the sample surface. Their subsequent influence on the measurement results of following correlation techniques was examined. To optimise the dataset quality, multi‐microscopy should be carried out in TUNA‐EBSD‐CL order, from the most to the least surface sensitive techniques. With the optimised multi‐microscopy measurement order on a CIGS absorber, we directly correlated the local changes in electrical and opto‐electronic properties with the microstructure of grain boundaries (GBs). The described methodology may also provide insightful concepts for applying other AFM‐SEM‐based multi‐microscopy on different semiconductor materials.

## 1 INTRODUCTION

Multi‐microscopy – also known as correlative microscopy – is a widely used characterisation approach in many materials and biology research fields. Specifically, multi‐microscopy involves applying several complementary microscopy techniques to the same region of interest to study various aspects of the material properties.^1^ This site‐specific correlative characterisation strategy can overcome the limitations of individual techniques and allows for the correlation of different material properties, such as structural, compositional, electrical, and optoelectronic properties. The cross‐verifying observation with different techniques can improve the reliability of datasets and may reveal important linkages between inter‐related properties.^2^ In recent years, multiple research studies have applied multi‐microscopy approaches, combining different characterisation techniques, to various semiconductor materials, including perovskite,^3^, ^4^ GaN,^5^, ^6^ GaAs,^7^ CdTe,^8^, ^9^, ^10^ Cu(In,Ga)S_2_ (CIGS),^11^, ^12^ etc., and have hence clarified the linkages between material properties and device performance.

Our research focuses on sulphide CIGS, a tetragonal chalcopyrite solar cell material. The wide and tuneable bandgap makes CIGS a promising candidate for the top cell of Si‐based tandem solar cell.^13^ Similar to its Se‐analogue Cu(In,Ga)Se_2_ (CIGSe), CIGS has demonstrated high energy conversion efficiency with polycrystalline absorber films, which are rich in grain boundaries (GBs).^14^, ^15^ These defects are nonradiative recombination centres, limiting the charge carrier diffusion length and the energy harvesting efficiency of solar cell modules.^12^ However, the high performance of polycrystalline CIGS solar cells suggests that the effect of GBs in CIGS may be more complicated. To fully understand the underlying physics and facilitate future material engineering, further research on the GBs in CIGS is needed. Since the behaviours of GBs may be influenced by several factors, such as orientation and composition, multi‐microscopy, which allows direct correlation between different properties, is a suitable research strategy.

Although the concept of multi‐microscopy is quite simple and straightforward, its execution is often troublesome and requires a good understanding of both techniques and materials. Several papers have reported the application of various atomic force microscopy (AFM), scanning electron microscopy (SEM) and transmission electron microscopy (TEM) based techniques to study various properties, including electrical, opto‐electronic, compositional and structural properties, of GBs in CIGS or similar material, such as Cu(In,Ga)Se_2_.^16^, ^17^ Limited research has reported the correlation of SEM and/or TEM observations.^12^, ^18^ However, the correlation across AFM and EM in CIGS has not been reported so far. In one side, correlating the same position across different machines in microscopic scale is difficult to achieve. In another side, some AFM‐based measurements, especially some electrical AFM modes, are highly sensitive to measurement conditions and sample surface status, which may be influenced by other measurements or treatments during sample preparation. It is meaningful to incorporate AFM‐based electrical techniques into multi‐microscopy, as electrical AFM modes can provide additional valuable information about materials, such as conductivity and surface potential, which may be difficult to measure with EM. To achieve effective multi‐microscopy with surface sensitive AFM techniques, optimising the measurement procedure is essential.

In this paper, we present a multi‐microscopy study combining tunnelling current atomic force microscopy (TUNA), cathodoluminescence spectroscopy (CL), and electron backscatter diffraction (EBSD). The first section focuses on the optimisation of the multi‐microscopy measurement routine on a representative CIGS sample. Optimising the measurement routine to minimise each techniques impact on the following techniques is crucial for high quality multi‐microscopy measurements as an improper measurement order may not only introduce contamination on sample surface but may also strongly influence the quality of subsequent measurement results. In section two, an example correlative TUNA‐EBSD‐CL study on a polished CIGS absorber, measured with an optimised multi‐microscopy routine, is given. Our approach allowed us to directly correlate the electrical and opto‐electronic properties of a solar cell absorber with its microstructure. We find, from our EBSD results, that we can classify GBs in CIGS into two distinct types, twin boundaries (TBs), and random high angle grain boundaries (RHAGBs). Correlating TUNA, EBSD, and CL we find that RHAGBs show low electric conductivity and strongly inhibit radiative recombination activity when compared to their adjacent grains. TBs on the other hand were observed to be mostly indistinguishable from the grain interior (GI) in the CL and TUNA signal, with a few exceptions that have a similar impact as RHAGBs.

## 2 METHODS

### 2.1 Sample preparation

The CIGS sample was fabricated on a Mo‐coated soda lime glass substrate using a 3‐stage co‐evaporation.^14^ The first stage was ∼260°C and the second and third stage was ∼570°C. The process was completed under a high sulphur partial pressure condition. The substrate kept constant rotation with 4 rpm during the growth.

**FIGURE 1:**
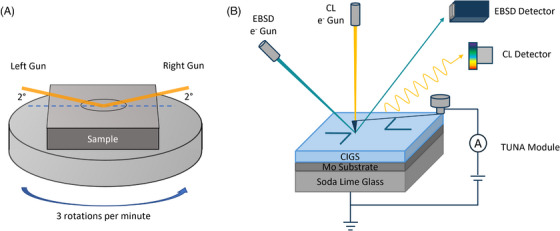
(A) Setup of sample polishing by broad ion beam. (B) Schematic shows the correlative TUNA‐EBSD‐CL measurements. In EBSD measurements, the sample was tilted 70° relative to the incident electron beam to maximise the collection of backscatter electrons by EBSD detectors. In both TUNA and CL measurements, the sample was placed horizontally.

To eliminate the as‐grown surface features and improve surface smoothness for TUNA and EBSD measurements, the sample was polished using a Gatan broad ion beam system. The sample was first polished by a 4 kV Ar ion beam for 45 min, followed by 15 min gentle polishing at 1 kV. All the polishing was completed at a temperature of 80K to minimise the formation of by‐products, such as Cu or CuO (Cu‐based) particles, under a high‐energy ion beam.^19^, ^20^ During the polishing, the sample stage was constantly rotated with a speed of 3 rotations per minute and the ion beam was tilted 2° relative to the sample surface, as shown in Figure 1A. The sample was kept in vacuum or under a nitrogen atmosphere between the polishing and the TUNA measurements, to minimise the potential influence of sample oxidation.

### 2.2 Characterisation techniques

The measurement setup for all three different measurements is listed below. The final correlative dataset was acquired in the order TUNA‐EBSD‐CL on the same region of interest, as shown in schematic Figure 1B.

TUNA measurements were carried out using a Bruker Dimension Icon AFM equipped with a Platinum‐Iridium‐coated SCM‐PIC‐V2 tip. The tip is scanned in contact mode over the sample surface. The sample is negatively biased relative to the conductive tip and the measurement was performed under ambient condition. The minimum sample bias to maintain a stable tip‐sample current flow across the map is applied in TUNA measurements. The surface topography and the electrical properties of sample were acquired simultaneously using a Bruker Peakforce TUNA module. The topography and electrical data were processed and analysed with using Bruker Nanoscope Analysis software and an open‐source Python toolbox PySPM.^21^

EBSD data were acquired using a Zeiss Gemini SEM equipped with an Oxford Instrument HKL Symmetry S3 EBSD detector. Kikuchi pattern indexing was achieved using the Oxford Instrument HKL AZtecHKL 4 software and a zinc blende lattice with lattice parameter of *a*_0_ = 0.558 nm. The cubic lattice was applied to eliminate the influence of pseudo‐symmetry on Hough‐indexing results. The potential influence of indexing lattice and pseudo‐symmetry on EBSD indexing results is discussed in detail in Supplementary Information. An acceleration voltage of 15 kV, a pixel step size of 35 nm, and 30 ms pixel dwell time were used for the EBSD acquisition. The EBSD results were processed and analysed by an open‐source MATLAB toolbox MTEX.^22^ An open‐source Python toolbox, Kikuchipy,^23^ was used for Kikuchi pattern analysis.

Hyperspectral room temperature CL measurements were performed by using an Attolight Allalin 4027 Chronos dedicated CL‐SEM. An acceleration voltage of 3 kV, a probe current of 1.25 nA, 50 µm aperture size, and 500 ms per pixel exposure time were used during the CL measurement. The CL data was processed and analysed with the open‐sourced python toolbox Hyperspy^24^ and Lumispy.^25^

### 2.3 Correlation

**FIGURE 2:**
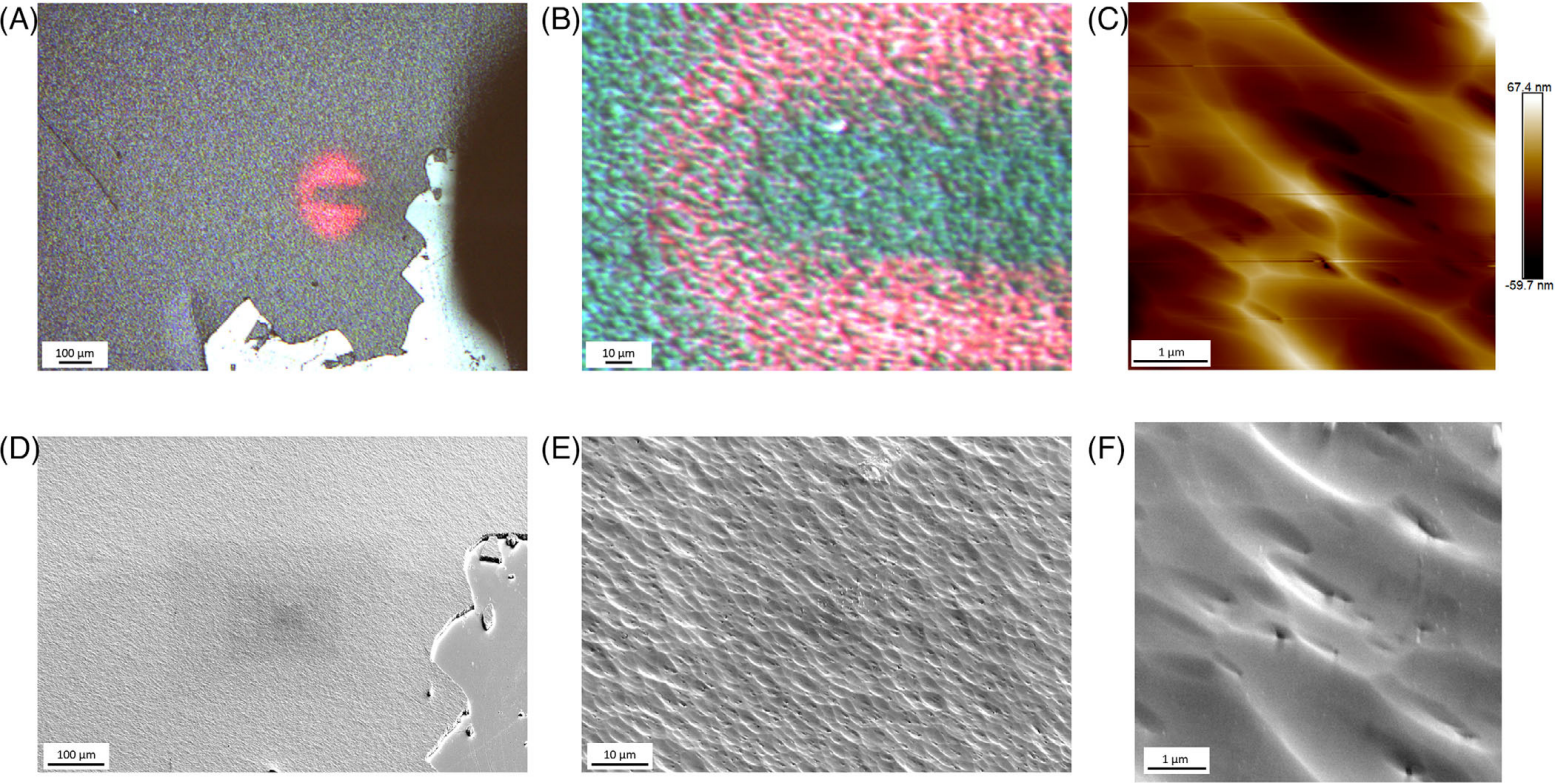
(A, B) Zoomed‐out optical microscopy images at AFM mapped region, which is shown in (C). Red regions in optical microscopy images come from laser light shooting on the apex of cantilever. By using the optical microscopy images as reference, the same region with different field of views (D–F) is found in SEM.

To facilitate the finding of region of interest under different microscopies, we created L‐shaped markers on the sample by scratching with a blade, as shown in Figure 1B, which may act as a simplified coordinate system. The characterisations were carried out mainly adjacent to the markers. After the completion of AFM‐based characterisation, a series of optical microscopy images around the AFM mapped area were taken by using the optical microscope installed in AFM, as shown in Figure 2A and B. Then the sample was moved to EBSD‐SEM and CL‐SEM with the same sample imaging orientation. By comparing the feature shown in optical microscopy images and AFM maps with the topography under SEM, the specific region of interest can be correlated in SEM. An example correlation process between AFM and SEM is shown in Figure 2.

## 3 RESULTS AND DISCUSSION

### 3.1 Optimisation of TUNA‐EBSD‐CL multi‐microscopy procedure

Depending on the sample stability and the operational parameters of the experiments, many characterisation techniques can alter the material to varying extents.^26^, ^27^, ^28^ In a correlative measurement, potential changes to the sample can strongly influence the results of subsequent measurements. Therefore, to optimise the multi‐microscopy procedure, it is crucial to understand the influence of different characterisation techniques on the sample and on how changes affect other involved techniques.

### 3.2 Influence of TUNA and SEM‐related technique on sample surface

Several studies have reported sample damage in the form of oxidation caused by TUNA or conductive AFM under ambient conditions.^28^, ^29^, ^30^ If a positive sample bias is applied, the conductive tip may effectively act as a cathode, and the water film on the sample surface may dissociate and behave as an electrolyte, leading to electrochemical oxidation of the sample surface.^31^ This process can be minimised with a negative sample bias, but oxidation via this or other mechanisms may still occur if improper experimental parameters, such as very high bias and long contact time, are used.^32^ As a product of a surface chemical reaction, the oxide can influence local chemical composition to some extent, which will add extra uncertainty to multi‐microscopy operation and results. Oxide particles can also interfere with the tip‐sample interaction in AFM‐related characterisation and influence the electron beam‐sample interaction in SEM‐related measurements. Depending on the operation parameters and interaction volume, the local measurement results can be strongly deteriorated. In addition, some large oxide particles may significantly disrupt local surface smoothness, acting as a barrier to block the pathway of backscatter electrons. Therefore, the oxide particles will not only deteriorate local area results, but also lower the data quality from the closely surrounding area.

**FIGURE 3:**
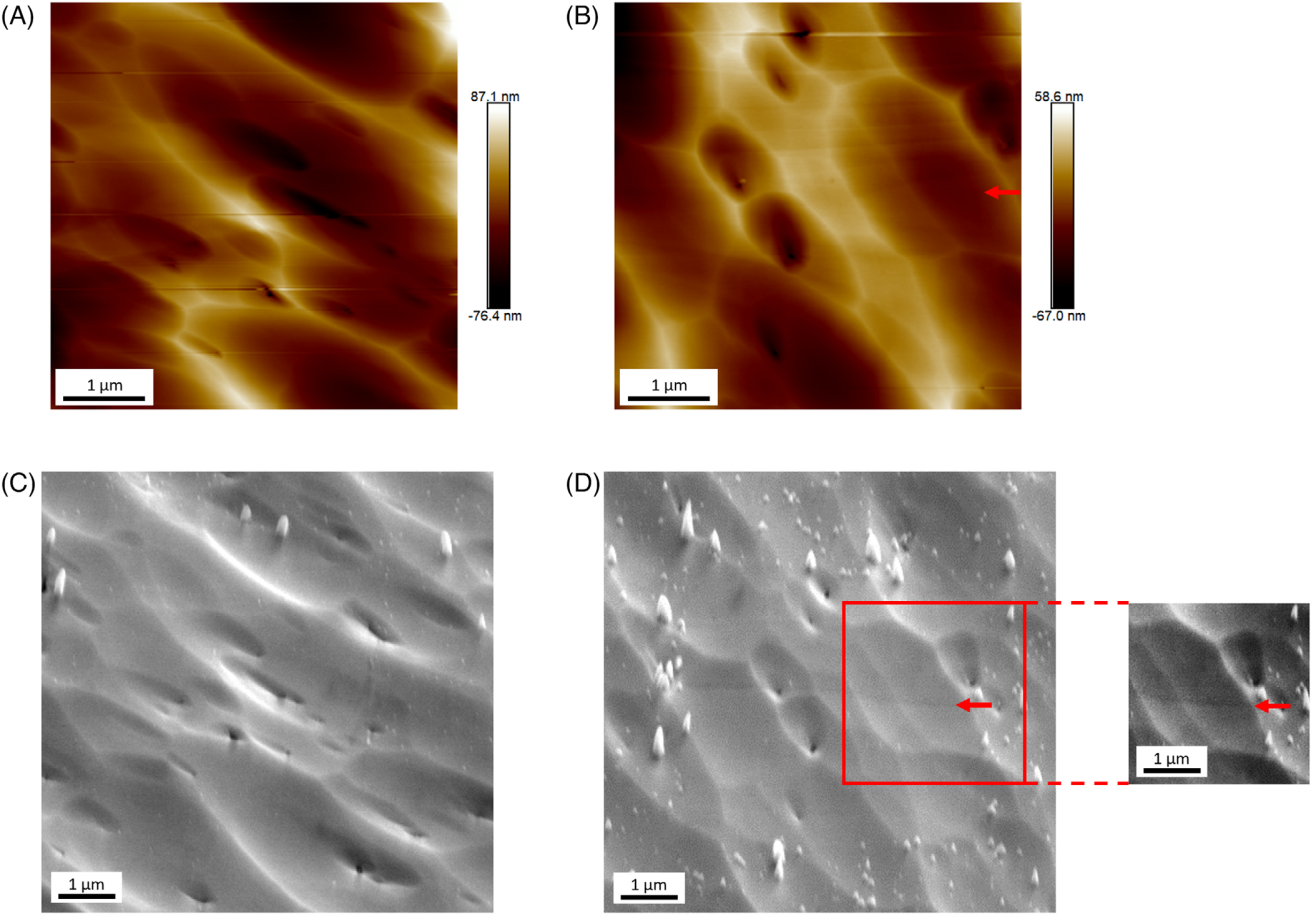
To test the influence of TUNA on surface status, a series of measurements in AFM‐TUNA‐SEM order was completed on two different area. (A) AFM and (C) SEM images of TUNA mapped area without topography change; (B) AFM and (D) SEM images of TUNA mapped area with minor TUNA‐induced topography change. The protrusion distributed in SEM image may be due to the aggregation of Cu‐based particles or other contaminations under electron beam exposure. The red arrow points out an area with contrast changes, which stands for weak topography change caused by high local current flow during TUNA measurement. For better comparison, a small area in (d) is cropped out and modified with brightness and contrast.

To avoid oxide formation during TUNA measurements, we applied the minimum negative sample bias necessary to maintain a stable TUNA current flow. Depending on the tip status and sample morphology, oxidation may still occur occasionally. To check for possible sample damage by TUNA, we carried out AFM topography measurements prior the TUNA mapping and SEM measurements after the TUNA mapping. Figure 3A and b shows the AFM images on two clean areas prior TUNA mapping. The dune‐like topography is mainly caused by the broad Ar ion beam polishing and few holes may linked to some cavities in absorber.^33^, ^34^ Enlarged SEM images on the corresponding area after TUNA mapping were shown in Figure 3C and D. In general, no obvious surface difference can be found between AFM maps and corresponding SEM images, indicating that the oxidation has been minimised by using suitable TUNA settings. Some minor topography change was still observed, as indicated by the red arrow in Figure 3B and D. The topography change with TUNA measurement may link to the sudden changes in tip‐sample contact and current flow density at some pits. In addition, it is worth noting that there is a frame of particles around the mapping area, especially in Figure 3D. The formation of protrusions may be due to the aggregation of Cu‐based particles or other contaminations under electron beam exposure. It is also possible that the AFM tip swept away local dust particles during AFM and/or TUNA measurements, leaving a cleaner area with less contamination.

Electron radiation may influence sample in many ways, such as hydrocarbon contamination, electron beam heating, radiolysis of materials, etc.^27^ Considering the relatively mild acceleration voltage employed in SEM (up to 30 kV) and low electron sensitivity of CIGS, the dominating impact of electron irradiation in our study should be hydrocarbon contamination, as shown in Figure 2D. The contamination may result from electron beam induced polymerisation of carbon rich organic molecules left on sample surface during sample preparation or from the residual gas atmosphere in the microscope.^35^ The thickness of hydrocarbon coating strongly depends on the operation parameters, such as dwell time/exposure time per pixel, acceleration voltage, measurement temperature, etc. Thick hydrocarbon layer can be extremely fatal for surface sensitive techniques, such as TUNA and EBSD, and may also reduce signal to noise ratio for other measurements with large interaction volume.

### 3.3 Influence of characterisation techniques on other techniques involved

**FIGURE 4:**
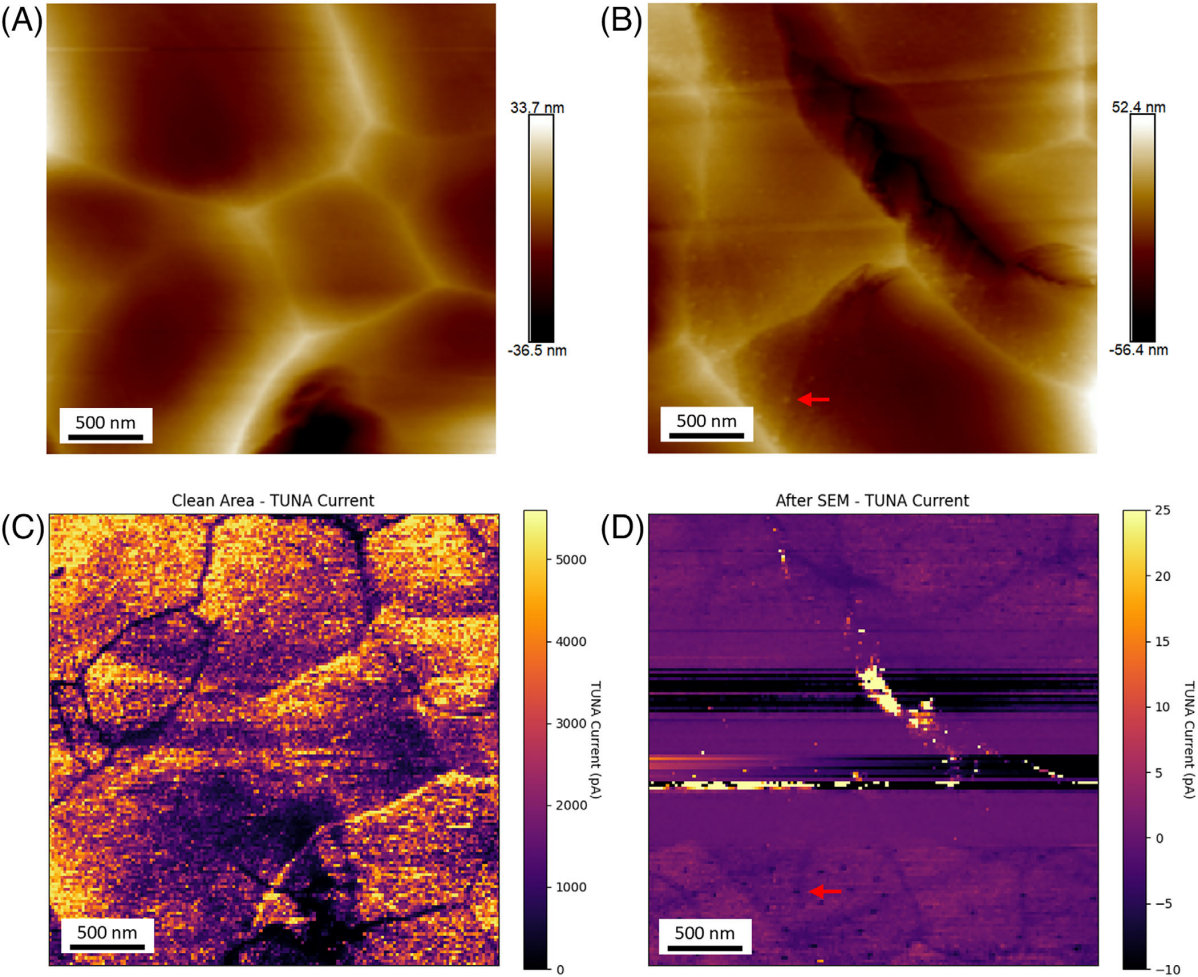
(A, B) AFM topography maps of clean area and after‐SEM area and (C, D) the corresponding TUNA current maps. Red arrows point out small particles formed under electron beam exposure.

We firstly examined the effect of electron beam exposure on TUNA results to assess the potential influence of SEM‐related techniques. To mimic the potential surface changes introduced by EBSD and CL, we scanned a freshly polished area by a 15 kV electron beam for 3 min in video mode. Two TUNA maps were acquired separately on a freshly polished area and on the area that had been exposed to the electron beam (the after‐SEM area). Figure 4a and b shows the topography of the non‐SEM area and after‐SEM area. A notable difference between these two areas is that the non‐SEM area shows much cleaner surface than the after‐SEM area. A considerable number of tiny particles with sizes around 5 to 10 nm was observed on the surface after SEM exposure, which may be caused by the aggregation of Cu‐based particles under strong electric field induced by the electron beam. Figure 4C and D shows the TUNA current maps acquired from the corresponding areas. In an ideal situation with minimal surface topography induced by ion beam preparation, the TUNA map should demonstrate any significant conductivity variation associated with the grain structure. Both TUNA maps in Figure 4 show grain‐like structures independent of most of surface topography, although occasional bright lines in the TUNA map are associated with particularly pronounced raised ridges in the topography. Overall, the data indicate the successful measurement of grain‐related TUNA variations. The after‐SEM area is observed to yield much weaker TUNA signals, compared to the clean area with similar level of bias applied, as indicated by the distinct colour bar between Figure 4C and D. The reduction of TUNA signals should be attributed to the hydrocarbon layer introduced by SEM imaging. We did find that the mid part of the after TUNA map has exceptionally strong TUNA currents compared to the rest of the map. However, the distinct high current area should be attributed to the sudden tip‐sample interaction change at abrupt topographic features, instead of the meaningful grain structure. Therefore, that specific region should be excluded when making comparison between non‐SEM and after‐SEM maps. Additionally, the tiny Cu‐based particles shown in topography were also observed to impact the TUNA maps resulting in nearly no TUNA signal. These tiny particles may interrupt the tip‐sample interaction and block the local current flow, leading to artefacts in the TUNA map.

It is worth noting that the actual parameters used in EBSD and CL measurements can be much more aggressive in comparison to those used in the experiment shown here. As described in Section 2, we applied a 3 kV electron beam with 500 ms exposure time per pixel in CL measurement and a 15 kV electron beam with 30 ms dwell time per pixel in EBSD measurement in correlative measurement. The resulting acquisition time for either EBSD or CL will be much longer than 3 min and will likely lead to a thicker hydrocarbon coating. Additionally, the focused electron beam used in EBSD and CL may cause stronger local sample heating and worse the hydrocarbon contaminations compared to video mode.

Next, we then focus on the influence of TUNA and CL on EBSD measurements. The underlying working principle of EBSD is the extraction of crystal orientation information by indexing backscatter electron diffraction pattern or Kikuchi pattern. Due to the small depth of interaction volume of backscatter electrons, EBSD is a surface sensitive technique. The estimated depth of the interaction volume for EBSD in Si using a 20 kV electron beam is less than 40 nm.^36^ For CIGS, a much denser and also polycrystalline material, the interaction depth may be 20 nm or even smaller. Subtle topographic changes and contamination can influence the quality of Kikuchi patterns, and hence affect the accuracy of later indexing. To examine the influence of different characterisation techniques on EBSD, we used the band contrast (BC) of EBSD Kikuchi pattern as the figure of merit of pattern quality. The BC of the Kikuchi patterns was extracted by an open‐sourced python toolbox, Kikuchipy,^23^ using the power spectrum of the Kikuchi patterns after Fast Fourier transform.^37^ By comparing the contrast of high frequency components, the Kikuchi bands with sharp intensity change, and low frequency components, the background with gradual intensity change, the signal‐to‐noise ratio can be computed. In general, a high signal‐to‐noise ratio Kikuchi pattern will have a BC close to unity.

**FIGURE 5:**
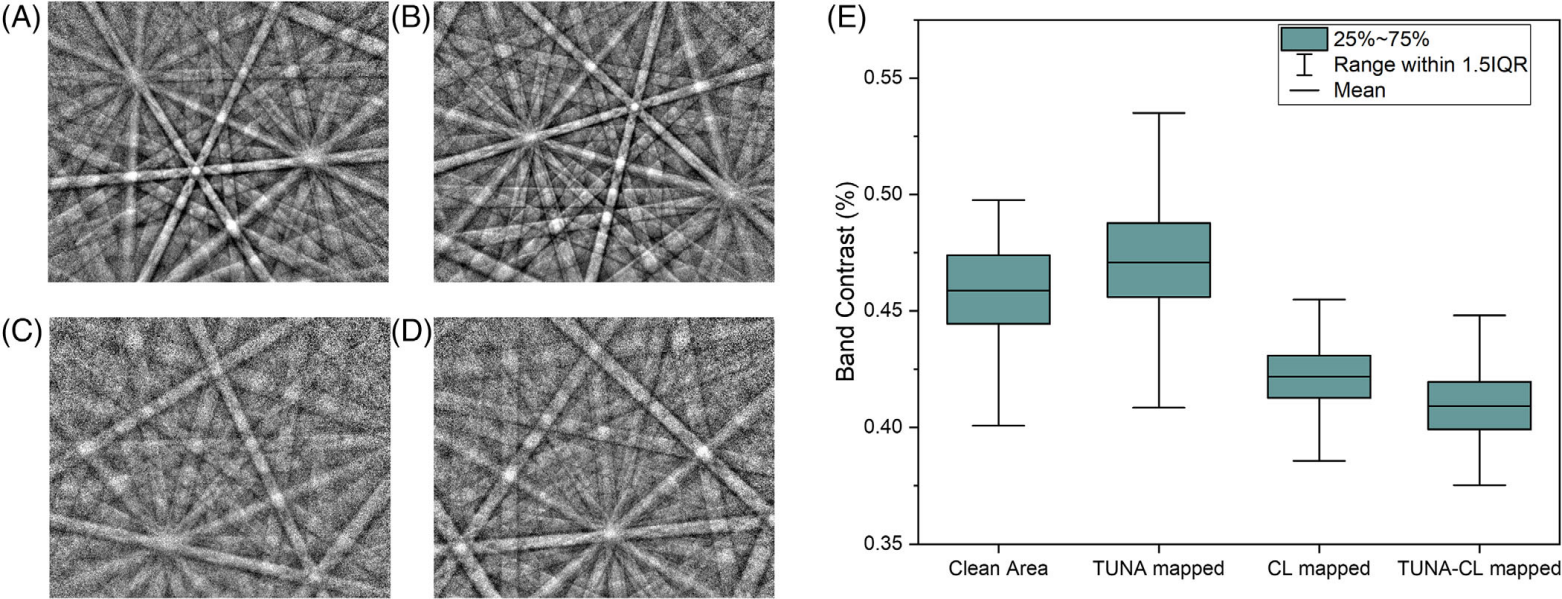
Example Kikuchi pattern and the corresponding band contrast of example Kikuchi pattern acquired from (A) a clean area, (B) a TUNA mapped area, (C) a TUNA‐CL mapped area, and (D) a CL mapped area. (E) The average band contrast of 3600 EBSPs from each region.

Figure 5A–D shows example Kikuchi patterns acquired from four different regions with the exact same measurement parameters. The average BC of EBSP from four regions were computed and shown in Figure 5E. The clean area pattern is used as a reference for a standard EBSD measurement result (Figure 5A). The TUNA mapped area (Figure 5B) has a high‐quality Kikuchi pattern with BC similar to the clean area. In contrast, both the CL mapped area (Figure 5C) and TUNA‐CL mapped area (Figure 5D) show much blurrier Kikuchi patterns and much lower BC, close to 0.4. An approximately 10% difference is found between CL mapped and non‐CL mapped area. The comparison may suggest that the impact of TUNA on EBSD measurement is subtle, while prior CL measurement may strongly deteriorate the quality of EBSD results.

**FIGURE 6:**
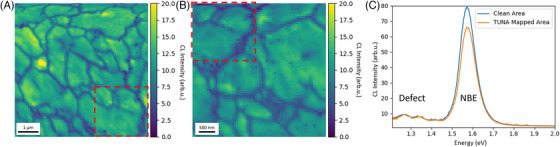
Panchromatic CL map acquired at (A) TUNA mapped area and (B) clean area; the red box highlighted the overlapped area between two maps. (C) Mean CL spectrum of corresponding maps.

Finally, to assess the potential influence of TUNA on CL, we carried out a correlative TUNA‐CL measurement and a standalone CL measurement for comparison. The panchromatic CL maps on a TUNA mapped area and on a clean area are shown in Figure 6A and B and the corresponding mean CL spectra are in Figure 6C. It is worth noting that the clean area CL was taken from an area immediately adjacent to that used for the correlative TUNA‐CL map and there is a region of overlap area, which is indicated by a red box. No distinctive difference can be observed between the two panchromatic maps, and both CL spectra show a similar ratio between the near band edge (NBE) peak and defect peak. For a quantitative comparison, we extracted the statistics of intensity, emission energy, and full‐width‐half‐maximum (FWHM) of the NBE peak by fitting the spectrum at each pixel with a Gaussian function. The statistics listed in Table 1 suggest a large degree of similarity between the CL results obtained from the clean area and the TUNA mapped area. Therefore, we may conclude that prior TUNA measurements do not exert a strong influence on CL results.

**TABLE 1:** Intensity, peak energy and FWHM of NBE peak of CL maps acquired from clean area and TUNA mapped area.

	CL intensity (arb.u.)	Emission energy (eV)	FWHM (eV)
Clean area	66.6 ± 25.6	1.580 ± 0.012	0.095 ± 0.012
TUNA mapped area	79.2 ± 29.6	1.580 ± 0.012	0.094 ± 0.012

From the comparative studies above, we conclude that for multi‐microscopy experiments, performing measurements in the order TUNA‐EBSD‐CL should provide the dataset with the best overall quality. In order to provide a more scientific basis for our practical guidance, we shall now discuss and compare the effective depth of interaction volume of different techniques, which is the origin of behaviour variations between techniques.

TUNA, as an AFM‐based technique, extensively relies on stable tip‐sample interaction. Any surface contaminations, or even topographic changes, may seriously interfere with the contact between the AFM tip and the sample. Therefore, the hydrocarbon contamination layer introduced by prior SEM measurements can significantly deteriorate the TUNA signal intensity by hindering the tip‐sample contact. EBSD and CL are both SEM based characterisation techniques utilising electron beam‐sample interaction to explore the material properties at various depths. However, due to the difference in working mechanism and setup as shown in Figure 1, the depth of interaction volume in EBSD should be significantly smaller than the 55 nm depth from which the CL signal can originate at 3 kV according to Monte Carlo Simulations.^38^ The acquisition of Kikuchi pattern therefore will be strongly influenced by the surface changes, such as surface oxidation and hydrocarbon contamination layers. In multi‐microscopy measurements, the optimised measurement order should go from highest surface sensitivity to lowest surface sensitivity, hence leading to the TUNA‐EBSD‐CL order that we suggest based on experimental results.

### 3.4 Correlative TUNA‐EBSD‐CL study of polished CIGS absorber

**FIGURE 7:**
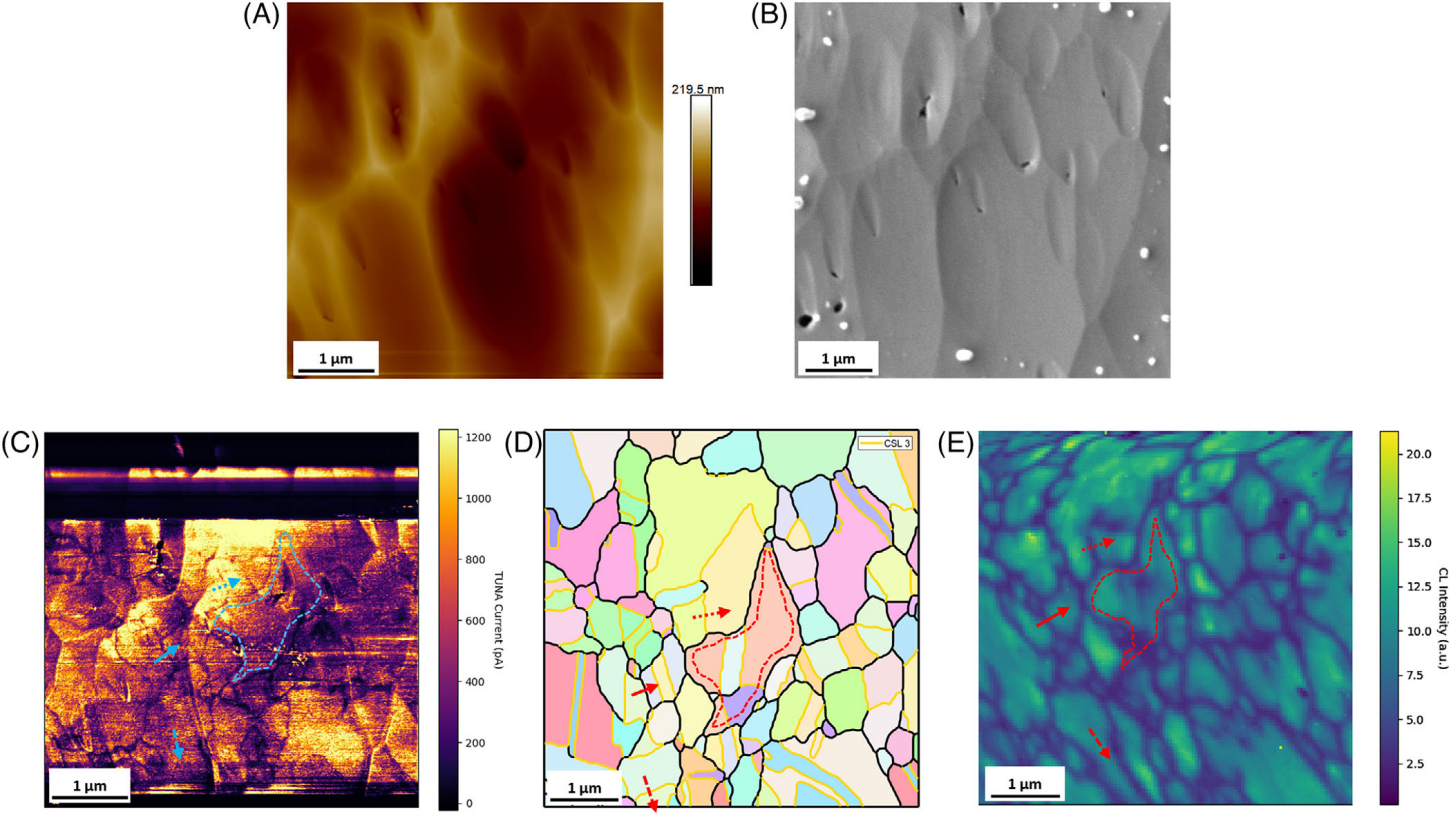
Example correlative TUNA‐CL‐EBSD datasets. (A) AFM topography and (B) SEM topography images, (C) TUNA current map, (D) EBSD grain orientation map, and (E) panchromatic CL map acquired on the same region of interest. To assist visualisation, a centre grain was highlighted by dotted line. TBs and RHAGBs are indicated in EBSD map by yellow and black lines, respectively. The solid line, the dashed line, and the dotted line shown in correlated maps indicated the position of example line scans on RHAGB, neutral TB, and dark TB. The corresponding TUNA and CL line scan profiles are shown in Figure 8.

In this section, we will demonstrate an example correlative TUNA‐EBSD‐CL study on a CIGS absorber. The topography obtained by AFM and SEM, TUNA current map, EBSD grain orientation and panchromatic CL maps from the same region of interest are all shown in Figure 7. The topography map exhibits a fairly smooth sample surface with small ridges and grooves after broad ion beam polishing. A similar topography pattern from the same region of interest is found in SEM, with some contamination particles surrounding it. Grain structure‐like patterns are found from TUNA maps, wherein grains with strong current flow were separated by nonconductive boundaries. TUNA signal variation can also be found at some of the ridges observed in topography, which may be caused by the abrupt change in tip‐sample interaction area arising from the topography variation. Apart from the occasional ridges, the majority of features in the TUNA map do not relate to features in the topography, suggesting that the topographic features after polishing may be largely unrelated to the grain structure. The EBSD map provides a confirmation of grain structure near the sample surface and can be used to compare with the pattern observed in TUNA map and later CL map. The CL map also exhibits a grain structure‐like pattern, with strong CL intensity within grain‐like features and weak emissions at boundary positions. Although the pattern in the CL map is distorted due to the strong charging effects during the long time required for map acquisition, the grain structure pattern still can be linked to the grain structure shown in TUNA and EBSD map. A centre grain is highlighted by dotted line to assist correlation.

**FIGURE 8:**
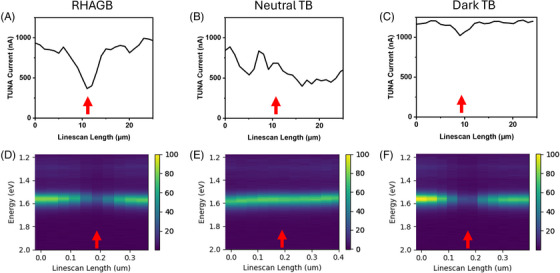
The TUNA and CL profiles extracted from the RHAGB, the neutral TB and the dark TB labelled in Figure 7. GB positions are highlighted by orange arrows.

With the TUNA‐EBSD‐CL multi‐microscopy, the electrical, opto‐electronic, and the structural information of CIGS can be directly associated. As labelled in EBSD map, GBs in CIGS are roughly divided into two types, the highly symmetrical TBs and RHAGBs. The grain structures shown in the TUNA and CL maps are similar to the distribution of RHAGBs shown in the EBSD maps. The RHAGBs are observed with much lower TUNA current and CL emission intensity than surrounding grains, suggesting that RHAGBs can suppress local electrical conductivity and radiative recombination activity. Example TUNA and CL line profiles on a RHAGB are shown in Figure 8A and D, respectively. It is worth noting that the CL emission also shows an energy shift between the two grains in the RHAGB line profile, which may be associated with the compositional variation of grains. The majority of TBs are found to have no impact on TUNA current above the noise level and not to impact the intensity of CL emission. As exhibited by the example TUNA and CL line profile in Figure 8B and E, no distinctive signal differences found between TB and GI. However, we did observe a few TBs with similar signal variation in TUNA and CL as RHAGBs, an example dark TB is labelled with dotted arrow and corresponding TUNA and CL line profile are shown in Figure 8C and F.

The variation in both the TUNA and CL observations may link to the atomic scale structure of the different GBs. The reduction in both the TUNA and CL signal intensity at RHAGBs may be attributed to the disruption of crystallinity and an agglomeration of point defects and impurities at the boundaries.^12^ The distinctive behaviour of neutral and dark TBs may associate with coherent and incoherent TBs. At a perfect coherent TB, two adjoining crystal grains are mirror images of each. In other words, the ideal coherent TB should be virtually free of dangling bonds and hence behave very similarly to pure grain.^12^ At an incoherent TB, the atomic arrangement on either side of the boundary may not match perfectly, giving space for the formation of point defects or dangling bonds, which may block the charge carrier movement and actively enhance nonradiative recombination.^39^

This example TUNA‐EBSD‐CL multi‐microscopy study on a CIGS absorber demonstrated the direct correlation of electrical and opto‐electronic properties on specific GBs, revealing the potential of the multi‐microscopy approach developed here. By extending the multi‐microscopy approach to multiple datasets, a statistically significant correlation between structure and properties can be achieved. We are looking forward to providing detailed analysis and comprehensive discussion about GB properties in CIGS absorber in a future study.

## 4 CONCLUSION

In conclusion, we present the optimisation of correlative TUNA‐EBSD‐CL measurement on freshly broad ion beam polished CIGS material. Both TUNA and SEM‐based techniques may lead to minor surface contamination. The impact of contamination on subsequent measurement was observed to be the strongest in TUNA measurements, and least important in CL measurements, whereas EBSD is in between these two extremes. The variation in sensitivity is due to the difference in the depth of interaction volume. Therefore, the multi‐microscopy should be carried out in the order TUNA‐EBSD‐CL, from the most surface sensitive to the least surface sensitive technique, to reduce any potential deterioration on results.

With the optimised multi‐microscopy, the influence of local microstructure on electrical and opto‐electronic properties was studied. Our findings demonstrate that all RHAGBs in our dataset strongly inhibit local conductivity and radiative recombination, while TBs were found to either behave similar to the surrounding GIs or occasionally to exhibit a strong reduction in CL intensity and TUNA currents. The structural difference between GBs is suggested to be responsible for the distinctive electrical and opto‐electronic properties of TBs and RHAGBs. This type of structure‐property correlation can be helpful to reveal the loss mechanism, such as nonradiative recombination and limitations to charge carrier transport.

We hope the optimisation described here will provide useful guidance for future multi‐microscopy in not only CIGS but also in the wider palette of semiconductor materials.

## CONFLICT OF INTEREST STATEMENT

The authors declare no conflicts of interest.

## Supporting information



Supporting Information

## Data Availability

The data that support the findings of this study are openly available in the University of Cambridge repository under https://doi.org/10.17863/CAM.113109.^40^
