# Supplementary material for: TUNA‐EBSD‐CL correlative multi‐microscopy study, on the example of Cu(In,Ga)S2 solar cell absorber
Source: J Microsc. 2025 Feb 2;298(1):106–17. doi: 10.1111/jmi.13393 (PMC11891954; doi:10.1111/jmi.13393)
Supplement: Supplementary file 1 — Supporting Information [file JMI-298-106-s001.docx]

**Supplementary information**

1. **Influence of indexing lattice and pseudo-symmetry on EBSD indexing results**

CIGS has a tetragonal lattice structure with the ratio of lattice constant, c/a ratio, only slightly deviated from 2. In other words, the CIGS tetragonal lattice is highly similar to the stacks of two cubic units. Therefore, the CIGS tetragonal lattice may share some characteristics of cubic structure. The phenomenon is known as pseudo-symmetry, and usually causes problems in EBSD indexing.^1^ For example, in CIGS material, certain orientations can resemble to each other after a 120° rotation around [221] axis direction, which refers to similar rotation around [111] axis direction in cubic lattice.^2^ Due to this rotational symmetry, different orientations can produce highly similar Kikuchi patterns with only subtle difference in some minor Kikuchi bands. Such a subtle difference is often not distinguished by Hough-based indexing in most commercial EBSD systems. The resulting EBSD map usually has a large amount of incorrectly indexed pixels.

In most previous chalcopyrite related EBSD study, a cubic zinc blende lattice was adopted to mitigate the indexing issue arising from pseudo-symmetry.^3–5^ The cubic indexing method can avoid noisy pixels, i.e. the mis-indexed pixels that otherwise appear in the tetragonal indexed map, producing sensible grain structure maps with correct identification of grain boundaries. However, the output grain orientation cannot reflect the true orientation of the material studied. To improve the validity of EBSD indexing and extract the correct crystallographic information from CIGS material, the dictionary indexing method can be a promising solution. Unlike Hough-based indexing which focusing on extracting certain major Kikuchi bands, the dictionary indexing method allows the comparison of the entire Kikuchi pattern.^6^ Therefore, dictionary indexing is a more robust method for noisy EBSP datasets with low signal to noise ratio and pseudo-symmetric materials.

To optimize the EBSD results for the multi-microscopy study of CIGS material, we compared the EBSD results indexed by Hough-based indexing with cubic and tetragonal lattice, and by dictionary indexing with tetragonal lattice. For EBSD dictionary indexing, we used an open-sourced Fortran-based software, EMsoft^7^. A CuInS_2_ master pattern with a tetragonal lattice structure at 15 kV acceleration voltage is generated. The detailed lattice constants are: a = b = 0.548 nm, c = 1.096 nm.

SI Figure 1 a) presents the EBSD pattern quality map which can provide an estimated indication for GBs. SI Figure 1 b), c), and d) show the EBSD results indexed by cubic and tetragonal Hough-based method, and tetragonal dictionary indexing method, respectively. Among the three indexed EBSD maps, the tetragonal Hough-based indexed map shows the noisiest EBSD result with a considerable number of mis-indexed pixels. Such a high density of mis-indexed pixels is hard to be processed in later data cleaning and may introduce fake GBs during GB computation. The tetragonal dictionary indexed map shows a significantly improved EBSD result. It is worth noting that some grains still encounter mis-indexing issues and may require further optimisation in the indexing method. The Hough-based indexing with cubic lattice shows the cleanest EBSD result. However, grain orientations shown in this map may not reflect true crystal orientations.


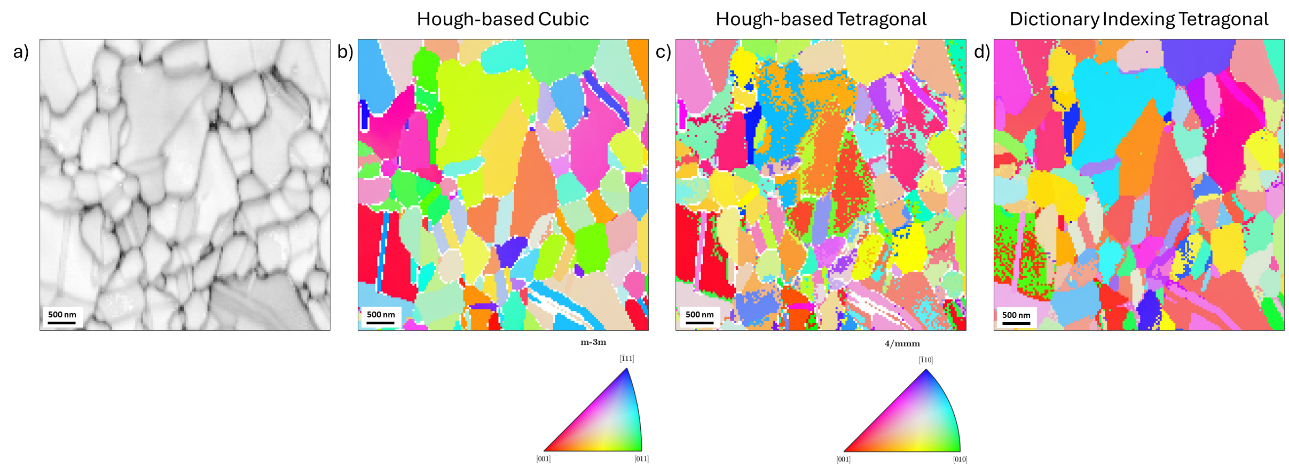


SI Figure 1 a) EBSD pattern quality map, b) EBSD results indexed by Hough-based indexing with zinc blende cubic lattice, c) EBSD results indexed by Hough-based indexing with tetragonal lattice, and d) EBSD results indexed by dictionary indexing with tetragonal lattice. All EBSD results are displayed as IPF z-direction.

The indexing method and lattice applied can influence the EBSD orientation results, and hence the computation of GBs. In cubic phase indexing, the GBs are categorized into RHAGBs and 60° twin boundaries (TBs), as shown in SI Figure 2 a). The EBSD result with tetragonal indexing shows RHAGBs, 60° TBs, and also 71° TBs, as indicated in SI Figure 2 d). Due to the presence of noisy mis-indexed pixels, the tetragonal indexed EBSD result may also have some GBs with misorientation angle at about 90°, which should be disregarded during analysis and multi-microscopy study. By comparing SI Figure 2 b) and e), it is obvious that the distribution of 60° TBs in the cubic indexing result is almost identical to that of 60° and 71° TBs in the tetragonal indexing result. Similarly, in terms of GB statistics (SI Figure 2 c) and f)), TBs accounts for about 40% of GBs in either cubic indexing or tetragonal indexing results.


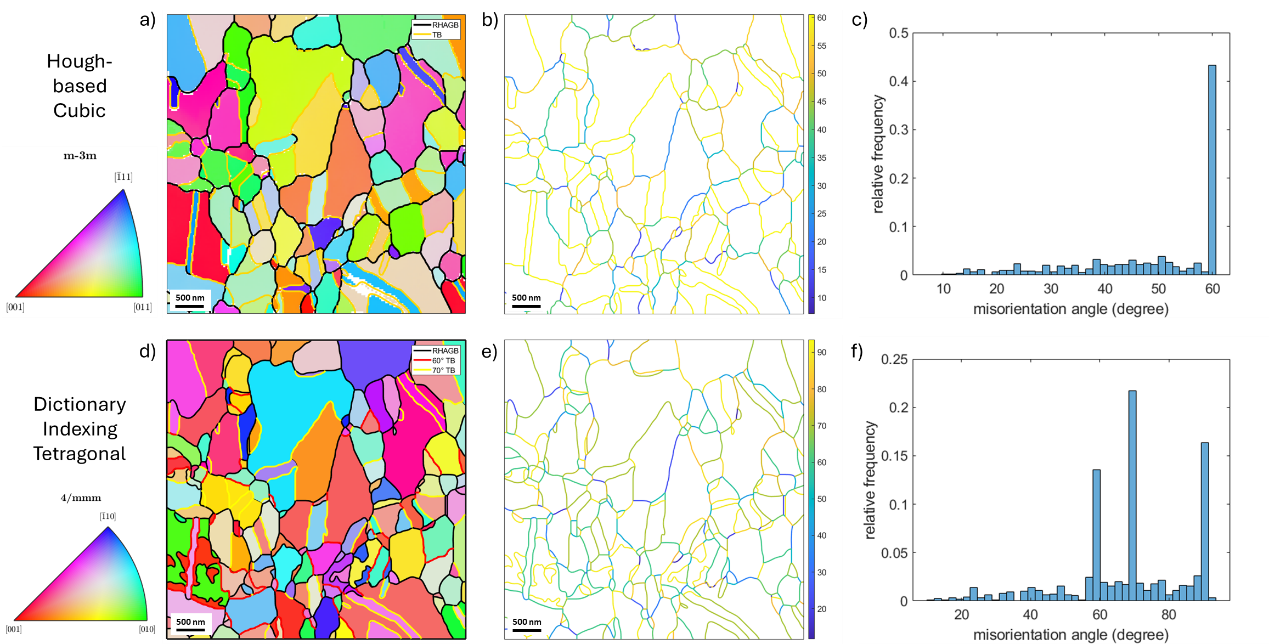


SI Figure 2 a) IPF z phase map with GB overlayed, b) GB misorientation angle distribution map, and c) GB misorientation statistics of EBSD results indexed by Hough-based method with cubic lattice; d), e), and f) show analysis for EBSD results indexed by dictionary method with tetragonal lattice.

The comparison study shows that the indexing method and the indexing lattice structure can influence the EBSD results obtained. The EBSD results still suffer from mis-indexing issues in some grains, even with the application of the dictionary indexing method. Regarding the GB analysis, both cubic indexing and tetragonal dictionary indexing results are qualified for the computation of RHAGBs and TBs.

By comparing TUNA and CL map (shown in main text) with all different EBSD results shown above, we did not find distinct inter-relationship between TUNA current/CL intensity and grain orientation. Because cubic indexing results can provide sensible GBs computation results and also avoid mis-indexed grains related boundaries which may cause confusions in correlation, we decided to present cubic indexing results in the correlation part of main text.

1. Pang, E. L., Larsen, P. M. & Schuh, C. A. Resolving pseudosymmetry in tetragonal ZrO2 using electron backscatter diffraction with a modified dictionary indexing approach. *J Appl Crystallogr* **53**, 1060–1072 (2020).

2. De Graef, M., Lenthe, W. C., Schäfer, N., Rissom, T. & Abou-Ras, D. Unambiguous Determination of Local Orientations of Polycrystalline CuInSe2 Thin Films via Dictionary-Based Indexing. *Physica Status Solidi - Rapid Research Letters* **13**, 1900032 (2019).

3. Hu, Y., Kusch, G., Adeleye, D., Siebentritt, S. & Oliver, R. Characterisation of the interplay between microstructure and opto-electronic properties of Cu(In,Ga)S2 solar cells by using correlative CL-EBSD measurements. *Nanotechnology* **35**, 295702 (2024).

4. Schwarz, T. *et al.* Correlative transmission Kikuchi diffraction and atom probe tomography study of Cu(In,Ga)Se2 grain boundaries. *Progress in Photovoltaics: Research and Applications* **26**, 196–204 (2018).

5. Müller, M., Abou-Ras, D., Rissom, T., Bertram, F. & Christen, J. Symmetry dependent optoelectronic properties of grain boundaries in polycrystalline Cu(In,Ga)Se2 thin films. *J Appl Phys* **115**, 023514 (2014).

6. De Graef, M. A dictionary indexing approach for EBSD. in *IOP Conference Series: Materials Science and Engineering* vol. 891 (IOP Publishing Ltd, 2020).

7. Jackson, M. A., Pascal, E. & De Graef, M. Dictionary indexing of electron back-scatter diffraction patterns: a hands-on tutorial. *Integr Mater Manuf Innov* **8**, 226–246 (2019).
